# Mechanism Identification and Kinetics Analysis of Thermal Degradation for Carbon Fiber/Epoxy Resin

**DOI:** 10.3390/polym13040569

**Published:** 2021-02-14

**Authors:** Han Li, Nasidan Wang, Xuefei Han, Haoran Yuan, Jiang Xie

**Affiliations:** College of Airworthiness, Civil Aviation University of China, Tianjin 300300, China; cauc_lihan@126.com (H.L.); nsdwang@163.com (N.W.); hanxuefei185@163.com (X.H.); yhr18041363619@163.com (H.Y.)

**Keywords:** thermal degradation, kinetics, gas analysis, carbon fiber/epoxy resin

## Abstract

For carbon fiber epoxy resin used in aerostructure, thermal degradation mechanism and kinetics play an important role in the evaluation of thermal response and combustion characteristics. However, the thermal decomposition process and mechanism are difficult to unify strictly due to the complexity of the components from different suppliers. In the present study, a product of carbon fiber epoxy resin made by AVIC (Aviation Industry Corporation of China) composite corporation is examined to identify its thermal degradation mechanism and pyrolysis products by measurements, including simultaneous thermal analysis, Fourier transform infrared spectroscopy and mass spectrometry, establish the kinetic model by Kissinger/Friedman/Ozawa/Coats-Redfern methods. The results show thermal degradation occurs in three steps under the inert atmosphere, but in four steps under air atmosphere, respectively. The first two steps in both environments are almost the same, including drying, carbon dioxide escape and decomposition of the epoxy resin. In the third step of inert atmosphere, phenol is formed, methane decreases, carbon monoxide basically disappears and carbon dioxide production increases. However, in air, thermal oxidation of the carbonaceous residues and intermolecular carbonization are observed. Furthermore, thermal degradation reaction mechanism submits to the F_4_ model. These results provide fundamental and comprehensive support for the application of carbon fiber epoxy resin in aircraft industry.

## 1. Introduction

Attributed to high resistance to corrosion, high specific strength and stiffness of FRP (Fiber Reinforced Polymer) material, its utilization in infrastructures and manufacturing is more and more popular. For example, concrete slabs with GFRP(Glass Fiber Reinforced Polymer) bars and hallow composite reinforcing system [1] and a novel prefabricated FRP composite jacket with an easy-fit and self-locking mechanical joining system [2] have been developed. In addition, carbon fiber epoxy resin has been widely used in the design and manufacture of aircraft load-bearing and functional structures. However, thermal behavior of FRP is completely different from that of aluminum, because it can soften, decompose and burn to release fumes, especially in fire, leading to threaten the safety of occupants. As a result, both the Federal Aviation Administration (FAA) and the European Aviation Safety Agency (EASA) require us to consider fire protection, flame retardancy, combustion product toxicity, and other thermal issues of composite aircraft structures in airworthiness certification [3]. For this reason, the generic material combustion computational model, ThermaKin [4,5,6,7] and a toxicity assessment model [8,9,10] have been developed by FAA. In addition, Tranchard [11] developed a material thermal response model based on SEMCEF for fire environments and validated it with the T700/M21 composite material used in Airbus 350. Li [12,13] also developed a thermal response model for composites in the fire environment based on ABAQUS.

Thermal response and combustion characteristics of carbon fiber epoxy resin in the fire environment mainly depends on the thermal decomposition of resin matrix. For this reason, Quintiere [14] has conducted reaction kinetic parameter tests, thermophysical property tests and flame retardant performance tests on fiber epoxy resins conforming to the Boeing Material Specification. Tranchard [15,16] also carried out similar tests on T700/M21 to obtain the reaction kinetic parameters of the material and to establish the reaction kinetic model, and to explore decomposition mechanism and the combustion products. Reaction kinetic parameters, including activation energy, reaction order and the pre-exponential factor, are typically obtained by the thermal analysis. The combination of thermal analysis and infrared or mass spectrometry analysis can more accurately analyze the reaction process and mechanism. Considerable progress has been made in modeling of kinetic [17,18,19,20,21,22,23], including multi- or single-step kinetic models that can be utilized in thermal–mechanical model [24], lightning strikes [25], fire behavior [11], curing process design, fiber recycling [26,27], etc.

Moreover, the complexity and even uncertainty of components of composites lead to a large amount of kinetics and mechanism research. Ahamad and Alshehri [28] studied the thermal degradation behavior of UF/epoxy resin blends (UFE) using a thermogravimetric analysis (TGA), Fourier transform infrared (FTIR) spectra and mass spectrometry (MS) to identify decomposition products such as CO_2_, CO, H_2_O, HCN, HNCO and NH_3_, and established a reaction kinetic model using the Coats–Redfern method to clarify the main decomposition mechanisms. Li and Wu [29] studied the pyrolysis performance and flame-retardant mechanism of flame retardant EP with respect to the synthetic amino resin intumescent flame retardant (AIFR), showing that AIFR advances the pyrolysis of EP, reduces the activation energy of pyrolysis, increases the residual carbon and decreases the intensity of the absorption peak in the infrared spectrum. Liu et al. [30] investigated the curing reaction of bisphenol A formaldehyde phenolic epoxy resin (bisANER) with diaminodiphenyl ether (DDE) and the thermal degradation properties of its cured products, and calculated the activation energy of bis-ANER/DDE non-isothermal curing reaction by the Kissinger method and Ozawa–Flynn–Wall method, also determined the thermal degradation mechanism under different atmospheres. Huang et al. [31] used thermogravimetric mass spectrometry to investigate the pyrolysis reaction behavior of the amino-phenolic resin. The kinetic parameters of the pyrolysis reaction of amino-phenolic resin were obtained by the kinetic analysis of the experimental data using the Coats–Redfern integration method, and the decomposition mechanism and pyrolysis products were clarified. Thomas et al. [32] used the standard Flynn–Wall–Ozawa (FWO) method to deduce the Arrhenius parameters from thermograms of biosourced substrates, hence, the energy of activation (E_a_) was obtained. Balart et al. [33] studied systematic kinetics of the thermal degradation of recycled ABS in inert atmosphere with using Flynn–Wall–Ozawa (FWO), the Kissinger–Akahira–Sunose (KAS) and the Starink methods, and suggested the degradation process of ABS takes place in a single-step process. Franco-Urquiza et al. [34] performed the thermogravimetric analysis at 5, 10 and 20 °C/min allowed determining the degradation kinetic parameters based on the Friedman and Kissinger models for neat polyester and nanocomposites, which showed that heating rates promoted an increase in the temperature degradation. Krawiec et al. [35] used a testing methodology to determine the toxicometric indicators on belts made of classically used fabric–rubber composite material, and evaluated the degradation kinetics of the polymeric belts by (TGA). Zhang et al. [36] investigated the effect of basalt fiber content in HDPE matrix composites on the thermal decomposition process using dynamic thermogravimetric analysis and utilized improved Coats–Redfern (C-R), Flynn–Wall–Ozawa (F-W-O), Friedman and Kissinger methods to ascertain the specific apparent activation energy (E_a_) of each component and composite material.

Therefore, for whether a new material or material modification, it is necessary to carry out thermal analysis, identify the thermal behavior or mechanism of degradation and establish the kinetic model. For carbon fiber epoxy resin used in aerostructure, it usually consists of a fiber, epoxy monomer, curing agent, catalyst and flame retardant, with complex composition and content. Therefore, the thermal decomposition process and mechanism of products from different suppliers are difficult to unify strictly. In this paper, for a new product of carbon fiber reinforced epoxy resin composite, BA3202, developed by AVIC (Aviation Industry Corporation of China) composite corporation for aerostructures, simultaneous thermal analysis (STA), FTIR spectra and mass spectrometry (MS) were conducted to obtain the reaction kinetic parameters, and a degradation kinetic model was established based on the Kissinger/Friedman/Ozawa/Coats–Redfern methods. Moreover, the thermal decomposition products of the material were identified to clarify the reaction mechanism. Compared to the published study in literature, a more comprehensive and thorough insight into thermal behavior of CFRP used in aerostructures is presented, and detailed data can support thermal response modeling, combustion modeling and toxicity assessment of material in fire.

## 2. Materials and Methods

**Figure 1:**
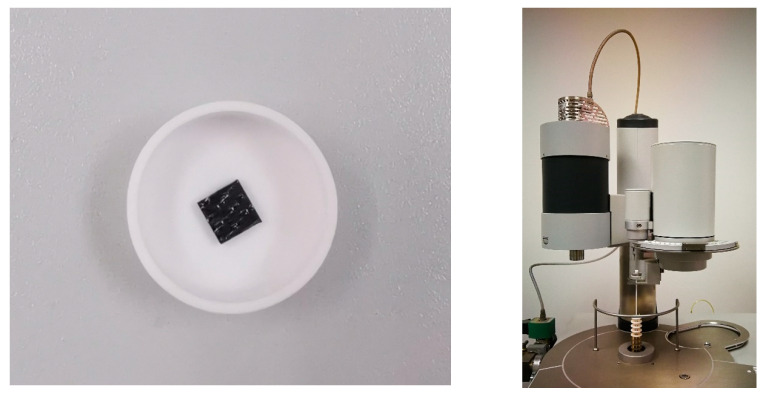
Specimen in the crucible and placement in the equipment.

Samples of carbon fiber reinforced epoxy resin matrix composite material used for aerostructures were provided by AVIC composite corporation (Beijing, China), in which matrix is mainly DGEBA. The preparation process was prepreg cutting-lay-up-autoclave molding–machining. The size of sample was 4 mm × 4 mm × 1.2 mm with the lamina thickness of prepreg of 0.15 mm, shown as Figure 1. Stacking sequence is [0/45/90/135]_s_ with the fiber volume of 54% and the density of 1.55 g/cm^3^.

Thermal analysis experiments were performed using the simultaneous thermal analyzer (NETZSCH STA 449 F3, NETZSCH, Selb, Germany), FTIR spectrometer (BRUKER VERTEX 80, BRUKER, Billerica, MA, USA) and mass spectrometry (NETZSCH QMS403C, NETZSCH, Selb, Germany) to investigate the mass changes (TG, Thermogravimetric), derivative of mass changes (DTG, Derivative Thermogravimetric) and gas composition (FTIR/MS), referring to the test standards [37,38,39,40]. The sample used in the simultaneous thermal analyzer was heated from room temperature to 1100 °C at the heating rates of 5, 10, 20 and 40 °C/min, under the atmosphere of air and inert gas, respectively, with the purge flow rate set to 20 mL/min. The primary aim of the TG analyses was to know the thermal degradation characteristics of materials, and to obtain adequate data to perform kinetic analysis in order to obtain the Arrhenius parameters. IR spectra were recorded in the spectral range of 4500–650 cm^−1^ with a resolution of 4 cm^−1^ and an average number of scans of 16. The intensities of 17 ions (*m*/*z* = 14, 16, 17, 18, 26, 28, 30, 42, 44, 46, 56, 60, 64 and 94) were determined according to the previous work [16,41].

## 3. Results and Discussion

### 3.1. Thermal Degradation

**Figure 2:**
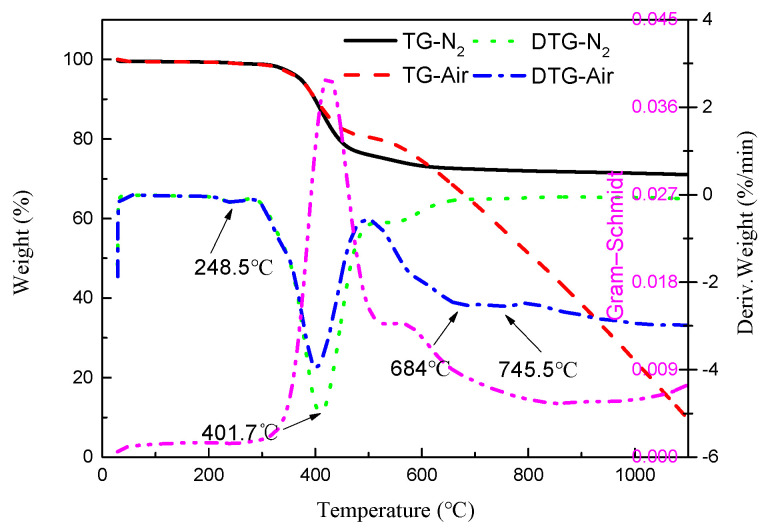
TG and DTG curves for the carbon/epoxy composite material carried out under inert and air atmosphere at 20 °C/min.

Figure 2 shows the TG and DTG curves of carbon fiber reinforced epoxy resin matrix composite heated from room temperature to 1100 °C at 20 °C/min under air and inert atmosphere, respectively. Under inert atmosphere, the TG curve shows three mass loss stages, corresponding to three DTG curve peaks. The first stage of weight loss ranged from room temperature to 286 °C with the mass loss of 0.69%, and the maximum weight loss rate was 0.17%/min with the peak temperature of 244 °C. At this stage, physical escape of low-molecular-weight gases occurs, with the drying effect caused by the temperature rise from room temperature, where water was evaporated rapidly, justified by mass spectrometry in Section 3.3.2. The same drying process is also observed in thermos-gravimetric analysis of epoxy resins by Ahamad [28] and Shao [42].

The second stage ranged from 286 to 515 °C with the mass loss of 23.29%, where the maximum weight loss rate was 4.99%/min at the peak temperature of 410 °C. This range is close to the pyrolysis interval of epoxy resin, where the ether bond is broken to generate BPA (Bisphenol-A), and the phenol comes from the further decomposition of BPA and/or the breakage of the C-C bond connecting the benzene ring after the breakage of unilateral ether bond on the epoxy resin [43], which is an obvious degradation behavior of the resin matrix. The weight loss of 4.54% in the third stage occurred in a wide temperature range from 515 to 1100 °C, which was relatively gentle, and the maximum loss rate was 0.64%/min corresponding to the peak temperature of 537.4 °C. According to the literature [44], the pyrolysis temperature of BPA epoxy resin is higher than 660 °C, so this stage was considered as further pyrolysis of the polymer [28].

Under the air atmosphere, the specimen was almost completely degraded with the mass loss of 90.64% reached at 1100 °C. The existence of four peaks in the DTG curve indicated that the weight loss process was divided into four stages, and the peak temperature was shown in Figure 1. The characteristics of the first weight loss stage in air atmosphere was almost consistent with that in inert gas, which was a typical drying stage. The peak temperature in the second stage was only slightly lower, but the pyrolysis interval was also similar to that under the inert atmosphere, indicating that the degradation of the resin matrix also occurred at this stage. Therefore, it showed that the reaction atmosphere has less influence on the degradation reaction of the resin matrix. However, the percentage of weight loss and weight loss rate in the inert atmosphere were greater.

The temperature range of the third stage was from 497 to 710 °C with the mass loss of 18.38%, the maximum weight loss rate of 2.53%/min and the peak temperature of 684 °C. Thermal oxidation of intermediate transient carbonaceous residues [16], which is a process of intermolecular cross-linking, carbonization, and further removal of small molecules, occurred at this stage [29]. The weight loss in the fourth stage ranged from 710 to 1100 °C with the mass loss of 52.65%; the maximum weight loss rate was 2.55%/min, and the peak temperature was 745.5 °C. This was due to the reaction of the carbon fiber reinforced material with oxygen and the nearly complete oxidative decomposition of the carbon fiber caused by prolonged heating, which was supported by literature [45].

### 3.2. Kinetic Model

**Figure 3:**
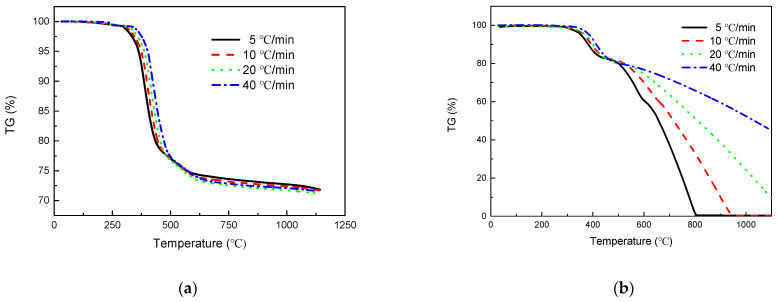

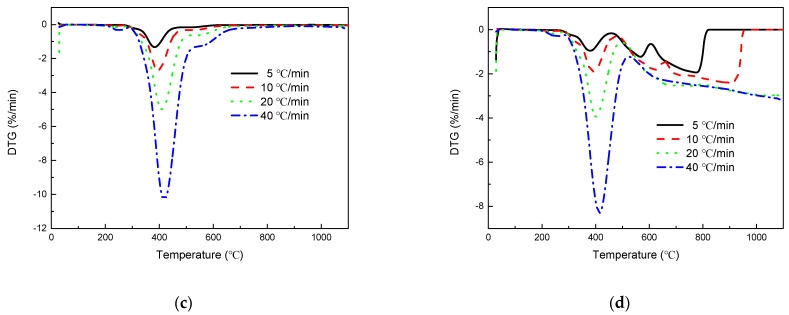
(**a**) TG curves and (**c**) DTG curves under inert atmosphere; (**b**) TG curves and (**d**) DTG curves under air atmosphere for T300/Epoxy composites at 5, 10, 20 and 40 °C/min.

Figure 3 shows the TG and DTG curves at different heating rates under inert and air atmosphere. Two inflection points appeared in the TG curves for different heating rates under inert atmosphere, which corresponded to the two peaks of the DTG curve. As the temperature rise rate increases, the weight loss curve shifts to the higher temperature region [46]. However, in air atmosphere, the effect of the temperature rise rate on weight-loss behavior appears more complex due to the oxidative decomposition of carbon fiber. The lower the temperature rise rate is, the longer the time is to reach the same temperature, and so the more sufficient the reaction is. As a result, the two lower heating rates (5 and 10 °C/min) make the temperature at which the weight loss rate reached the peak value lower, so that two more peaks could be seen below 1000 °C. However, under the heating rates of 20 and 40 °C/min, the degradation was not completed within 1100 °C, and no corresponding peaks were observed.

The different methods for solving the kinetic constants of reactions are based on the same basic theoretical formula derived from the Arrhenius formula, as shown in the following equation:

(1) $$\frac{d\alpha }{dt}=A\exp\left(\frac{-E_{A}}{RT}\right)f\left(\alpha \right),$$

where $\alpha =\left(M_{i}-M\right)/\left(M_{i}-M_{e}\right)$ is the conversion degree, $M_{i}$, M, $M_{e}$ are the initial mass, the mass of the specimen at the current moment, and the final mass at the end of the reaction, respectively. f(α) is a function of the kinetic mechanism, which is a function of the conversion degree, depending on the specific degradation mechanism. R is the ideal gas constant, with a value of 8.314 J/(mol K).

In this paper, the kinetic parameters of the reaction were solved by the Kissinger [47] differential method, the Friedman [20] differential method and the Ozawa [22] integral method, respectively. These three methods did not require the degradation reaction mechanism of the material to obtain the activation energy. As to calculate the reaction order, it can be assumed that the mechanism function can be expressed as follows: $f\left(\alpha \right)={\left(1-\alpha \right)}^{n}$. The Kissinger method [47] assumes the maximum reaction rate at the peak temperature. The Friedman method [20] assumes that the reaction kinetic constant *A*, *E_A_* and n are independent of the heating rate. While the Ozawa method [22] assumes that the conversion degree at the peak temperature at different heating rates is a constant (iso-conversion method). Additionally, the Coats–Redfern method was used to determine the reaction mechanism function. The data obtained under inert atmosphere are shown in the following Table 1.

#### 3.2.1. Kissinger Method

Differentiation on both sides of (1) yields the following equation:

(2) $$\frac{E_{A}\beta }{RT_{m}^{2}}=An{\left(1-\alpha_{m}\right)}^{n-1}\exp\left(\frac{-E_{A}}{RT_{m}}\right),$$

where $T_{m}$ is the peak temperature from the DTG curve, $\alpha_{m}$, is the peak conversion corresponding to $T_{m}$, which is obtained from the TG curve, and $\beta =\frac{dT}{dt}$ is the constant heating rate during the experiment. Kissinger considers $n{\left(1-\alpha_{m}\right)}^{n-1}$ is independent of β and its value is approximately equal to 1.

The activation energy can be obtained from the above equation by logarithm and derivation:

(3) $$\frac{d\left(\ln\left(\beta /T_{m}^{2}\right)\right)}{d\left(1/T_{m}\right)}=-\frac{E_{A}}{R},$$

**Figure 4:**
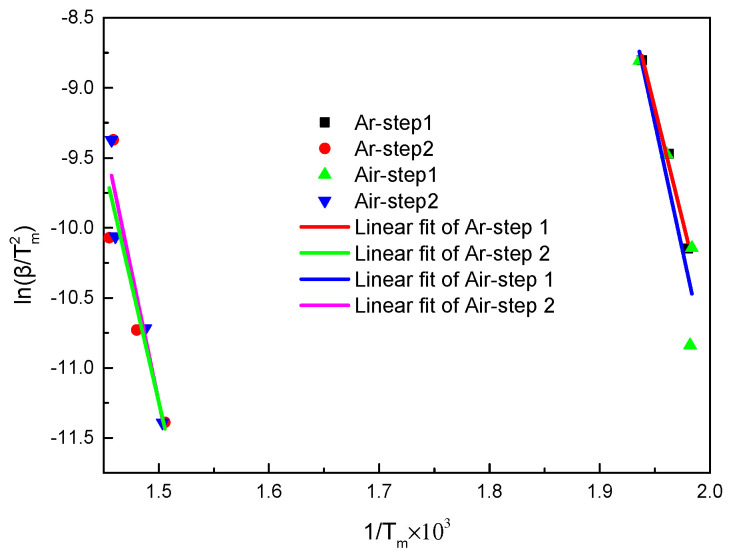
Plots of $\ln\left(\beta /T_{m}^{2}\right)$ − $1/T_{m}$ by Kissinger method under inert and air atmosphere.

The thermal weight loss process of carbon fiber reinforced epoxy resin composites under inert atmosphere has two main stages. As shown in Figure 4, the intercept and slope data are obtained.

The relationship between the pre-exponential factor *A*, and the slope and intercept of the curve is as follows:

(4) ln(A/(−k))=a,

When calculating the reaction order *n*, consider the mechanism function to be expressed as $f\left(\alpha \right)={\left(1-\alpha \right)}^{n}$ and *n* is calculated by the following equation:

(5) $$-n{\left(1-\alpha_{m}\right)}^{n-1}=1+\left(n-1\right)\frac{2RT_{m}}{E_{A}},$$

The results are shown in the Table 2 below:

#### 3.2.2. Friedman Method

Taking the logarithm of (1) gives the following equation

(6) $$\ln\left(\beta \frac{d\alpha }{dT}\right)=\ln\left(A\right)+n\ln\left(1-\alpha \right)-\left(\frac{E_{A}}{RT}\right),$$

where $\ln\left(\beta \frac{d\alpha }{dT}\right)=\ln\left(\frac{d\alpha }{dt}\right)$.

**Figure 5:**
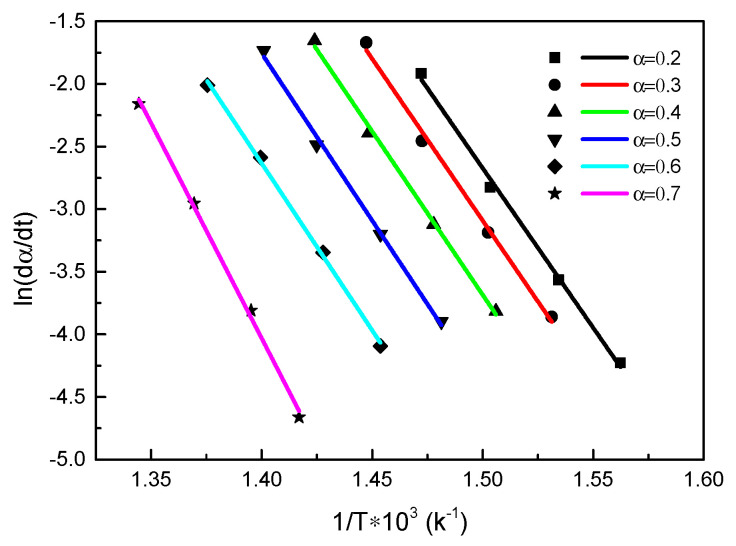
Plots of $\ln\left(\frac{d\alpha }{dt}\right)$-1/T by the Friedman method under inert atmosphere.

The relationship between $\ln\left(\frac{d\alpha }{dt}\right)$ and 1/*T* at different conversion degrees was calculated as shown in Figure 5.

**Figure 6:**
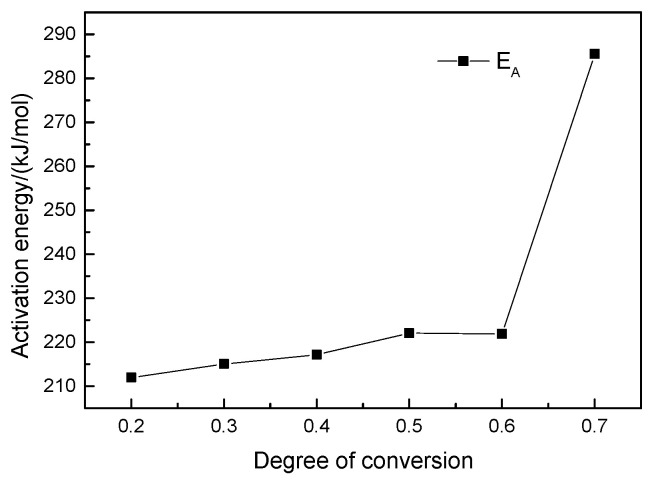
Activation energy as a function of fractional mass loss by the Friedman method under inert atmosphere.

Figure 6 shows a line chart of the activation energy with conversion degree, which illustrates that the activation energy is not a constant, but changes as the thermal decomposition reaction proceeds and the conversion degree increases, showing an overall upward trend. Therefore, the thermal decomposition reaction of carbon fiber reinforced epoxy composite under inert gas is a multi-step. Furthermore, the predominant factor was found to be 1.34287 × 10^14^.

#### 3.2.3. Ozawa Method

The activation energy *E_A_* was calculated by the following formula:

(7) $$E_{A}=-\frac{R}{0.4567}\frac{d\left(\log\beta \right)}{d(1/T)},$$

**Figure 7:**
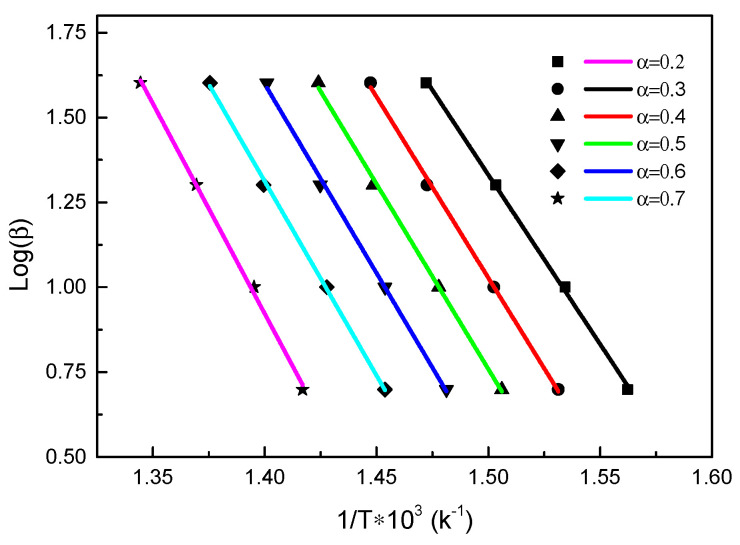
Plots of logβ-1/*T* by the Ozawa method under inert atmosphere.

The relationship between log *β* and 1/*T* was calculated for different conversion degrees as shown in the following Figure 7:

The pre-exponential factor was calculated by the following formula [48,49]:

(8) $$\log A=\log\beta +\log E_{A}+0.434E_{A}/RT-\log R-2\log T,$$

**Figure 8:**
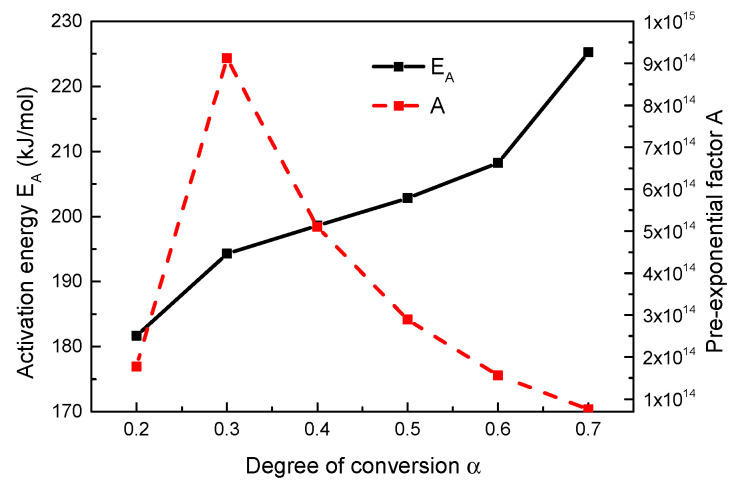
Activation energy and pre-exponential factor as function of fractional mass loss by Ozawa method under inert atmosphere.

At the same conversion degree, yielding the values of the corresponding pre-exponential factor for different heating rates, as shown in Figure 8. And the mean values were finally obtained. The activation energies obtained by Ozawa’s method were similar to those obtained by Friedman’s method, which verified the accuracy of the results.

#### 3.2.4. Coats–Redfern Method

Coats and Redfern assumed the function of the kinetic mechanism is $f\left(\alpha \right)={\left(1-\alpha \right)}^{n}$, and derived the following expression:

(9) ln(αT2)=ln(ARβEA)[1−(2RTEA)]−(EART),

where α in the logarithmic term of the left equation is the result of integrating 1/f(α) to α and then ignoring higher-order terms under the condition of low conversion rate after polynomial expansion. In order to study the kinetic model of thermal decomposition, assuming that the mechanism function is unknown, the alternative term to α in the logarithmic term is the integral form F(α) of 1/f(α), and considering that 2RTEA approaches zero, the formula is as follows:

(10) ln(F(α)T2)=ln(ARβEA)−(EART),

Different mechanism models were established by this method, as shown in Table 3.

The ln(F(α)T2) − 1/*T* curves for the conversion rates from 0.2 to 0.7 at different heating rates were made to obtain the activation energy values. Pearson’s correlation coefficients were calculated to measure the correlation between the variables, as shown in Table 4. Comparing the activation energy values in Table 4 with those calculated by the three kinetic methods in Table 1. The average E_A_ of F_4_ is the closest to the previous calculation, which was 211.691 kJ/mol. It is concluded that the pyrolysis reaction of carbon fiber reinforced epoxy resin composite in the inert atmosphere is the closest to that predicted by the F_4_ model. The expression of the mechanism function is: ${\left(1-\alpha \right)}^{4}$. After averaging the activation energies and pre-exponential factors of the four heating rates, the reaction mechanism model was obtained as follows

(11) $$\frac{d\alpha }{dt}=8.26\times 10^{11}\exp\left(\frac{-211691}{RT}\right){\left(1-\alpha \right)}^{4},$$

### 3.3. Gas Phase Analysis

#### 3.3.1. FTIR Analysis

The Gram–Schmidt spectrum under inert atmosphere in Figure 2 represented the FTIR absorption intensity of the escaping gas during pyrolysis of the material, showing that there were only two peaks at 425 °C and 557 °C, respectively, which were delayed compared to the corresponding DTG curve. This was due to the time delay between gas detection and gas generation in the FTIR spectrometer. The larger peak at 425 °C indicated that the amount of gas escaping at this stage was higher, and had a higher infrared extinction coefficient.

**Figure 9:**
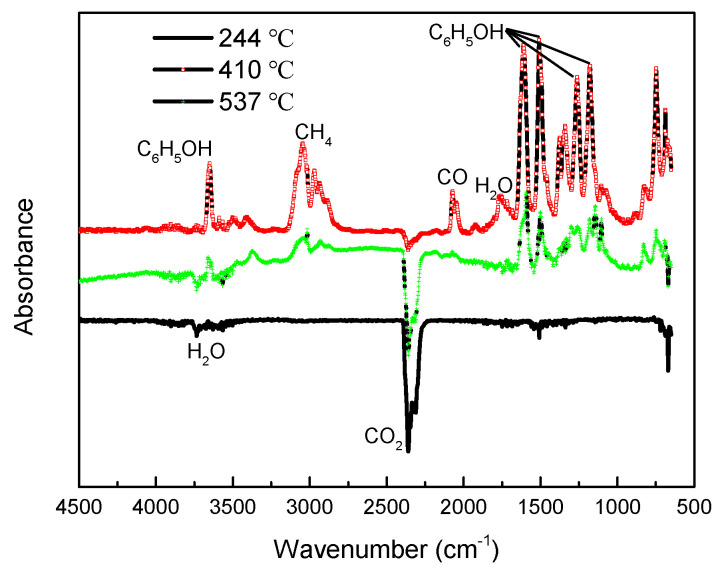
Spectra collected at 244, 410 and 537 °C of the degradation products of composites released during TGA (20 °C/min–inert atmosphere).

Figure 9 shows the infrared spectral data of the gas at the characteristic temperatures determined by the DTG curve. At the characteristic temperature of 244 °C, which was the first stage of mass loss, the products were carbon dioxide (CO_2_: 2300–2400 cm^−1^) and water (H_2_O: 3500–4000 cm^−1^). The same phenomenon is observed by Ahamad [28] in the study of urea-formaldehyde/epoxy resin blends. Thus, this stage was not only a drying stage, but also a physical escape of small molecule gases. Obvious degradation behavior of the resin matrix occurred in the second stage, corresponding to the infrared spectral data at 410 °C. Since the stretching vibration of saturated hydrocarbon C-H is usually close to the frequency absorption of 3000 cm^−1^, it is speculated that there was methane gas (CH_4_) according to the three peaks in the range of 2900–3170 cm^−1^: 3045 cm^−1^, 3016 cm^−1^ and 2970 cm^−1^. In addition to water and carbon dioxide, carbon monoxide (CO: 2000–2200 cm^−1^ with double peaks at 2048 cm^−1^ and 2070 cm^−1^) and phenol (main peaks of C_6_H_5_OH: 3650, 1608, 1510, 1260 and 1176 cm^−1^) were also observed. The above products were considered to be the chain scission and depolymerization reactions of BPA epoxy resins [31], where CO and CO_2_ are mainly derived from the ether group (R-O-R’), methyl group (-CH_3_), methylene group (-CH_2_) and other carbon-containing functional groups. At 537 °C, carbon monoxide almost disappeared and turned into carbon dioxide, while the production of the other gas products decreased compared with that at 410 °C.

#### 3.3.2. MS Analysis

The following gas molecules were detected by mass spectrometry at a heating rate of 20 °C/min under inert atmosphere: CH_2_ (*m*/*z* = 14), CH_4_ (*m*/*z* = 16), H_2_O (*m*/*z* = 18), C_2_H_2_ (*m*/*z* = 26), CO (*m*/*z* = 28), C_2_H_4_ (*m*/*z* = 28), C_3_H_6_ (*m*/*z* = 42), CO_2_ (*m*/*z* = 44), C_3_H_8_ (*m*/*z* = 44) and phenol C_6_H_5_OH (*m*/*z* = 94).

**Figure 10:**
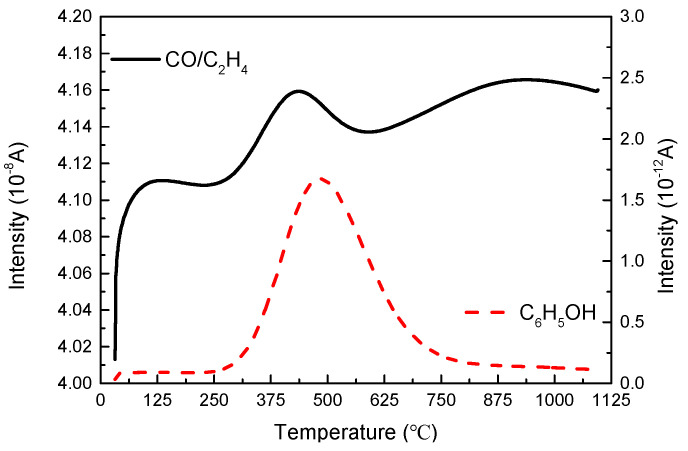
Intensity curves of *m*/*z* = 28 and 94 degradation products of composite released during TGA (20 °C/min-inert gas).

According to the order of magnitude of ion current intensity, the classification analysis was carried out from high to low. As shown in Figure 10, the left ordinate shows the mass spectra of gas products with a mass-to-charge ratio of 28, which was supposed to be CO and/or C_2_H_4_. Additionally, there were three peaks in the curve, which respectively occurred at 125 °C, 437 °C and 920 °C. The peak with the largest ion current intensity appeared between 300 and 500 °C, corresponding to the main decomposition peak in the DTG analysis, which was presumed to be the precipitation of CO gas, also observed by Tranchard [16] and Ahamad [28] and related to the certain amount of oxygen-containing groups in the polymer. The mass spectrum of phenol with the mass-to-charge ratio of 94 was shown on the right ordinate. Its precipitation range was 300–750 °C, which covered the second and third mass loss stages, also corresponded to the peaks of infrared spectrum at 410 °C and 537 °C.

**Figure 11:**
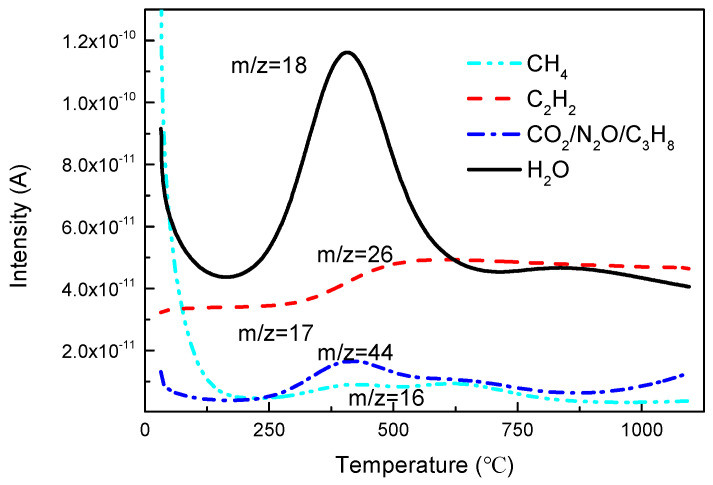
Intensity curves of *m*/*z* = 16, 18, 26 and 44 degradation products of the composite released during TGA (20 °C/min-inert atmosphere).

For the detection result of water molecules in Figure 11, a trough appeared before 250 °C, which corresponded to the first peak in the DTG curve, verifying that this was the drying stage. In addition, due to the pyrolysis reaction of the resin, the peak of water molecules appeared at 406 °C, which was ahead of the precipitation of CO, indicating that the decomposition of the epoxy resin would preferentially produce water molecules, but mainly precipitated CO. At this stage, the water produced by the thermal decomposition of epoxy resin mainly come from the breaking of the bonds of -OH functional groups.

For the gas mass-to-charge ratio of 44, CO_2_, N_2_O and C_3_H_8_ were considered. However, the peak of the curve occurred at 410 °C, ahead of 435 °C, which was the precipitation temperature of the CO peak. Considering the sequence of reactions, CO mainly comes from the reforming of the C-O group in the bisphenol A epoxy resin, and then combined with oxygen ions to form CO_2_. Therefore, it was speculated that the *m*/*z* = 44 mass spectrum curve was mainly represented by the C_3_H_8_ gas molecule. Since the reaction was carried out under an inert atmosphere, oxygen ions were supplied only by the material itself, which was also verified in the order of magnitude. It was speculated that the relatively small increasement of the amplitude of this curve afterwards might correspond to the formation of CO_2_.

The peak temperature of C_2_H_2_ gas molecules was close to the temperature of the third peak in the DTG curve, which was supposed to be the product of the second step of decomposition. It can be verified that the decomposition of carbon fiber reinforced epoxy resin matrix composite under inert atmosphere exhibits at least two steps (a major step and a secondary step).

#### 3.3.3. Effect of the Heating Rate on Gas Phase Products

**Figure 12:**
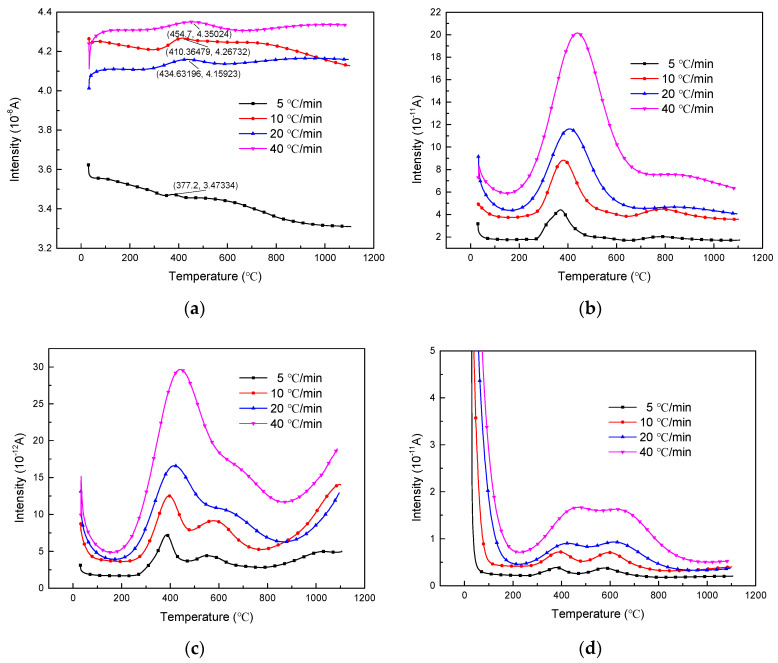
(**a**) CO, (**b**) H_2_O, (**c**) CO_2_ and (**d**) CH_4_ release against temperature with the heating rate during pyrolysis.

The increase of the heating rate leads to the phenomenon of thermal hysteresis, which is specifically manifested as the peak temperatures of CO, H_2_O, CO_2_ and CH_4_ ion flow all move toward the high temperature region with the increase of the heating rate. The production of H_2_O, CO_2_ and CH_4_ all increase with the increase of heating rate as shown in Figure 12, which indicates that faster temperature rise promotes the reaction. The production of CO had a relatively obvious weak value at the low heating rate, but had little change at the heating rates of 10 °C, 20 °C and 40 °C/min.

## 4. Conclusions

Thermal degradation of a new product of carbon fiber reinforced epoxy resin composite, namely BA3202, occurs in three steps under inert atmosphere, but in four steps under air atmosphere, respectively. The first two steps were almost the same. The first step is the physical changes, mainly water drying and carbon dioxide escape. The second step is the decomposition of the epoxy resin matrix, and the products are mainly methane, water, carbon monoxide, carbon dioxide and phenol. The third step in the inert atmosphere is the further pyrolysis of the polymer, in which phenol is formed, methane decreases, carbon monoxide basically disappears and carbon dioxide production increases. However, in air, the thermal oxidation of the carbonaceous residues and the intermolecular carbonization are observed. Furthermore, the last step in air is the oxidative decomposition of the carbon fibers. In addition, it is concluded that faster temperature rise can promote the reaction by the experimental comparison in different heating rates. These results provide a comprehensive and thorough understanding into the thermal behavior of CFRP in the thermal environment or fire, and can be used to support material selection and airworthiness certification of aviation structure.

Moreover, the activation energy value obtained by mechanism F_4_ is closed to that of the Kissinger/Friedman/Ozawa’s method. Therefore, it can be inferred that the thermal degradation reaction mechanism of CFRP for this study obeys the F_4_ model, which indicates the order of reaction is 4. As a result, the kinetic equation is obtained to support the application of carbon fiber reinforced epoxy matrix composite, such as modeling and assessment of thermal protection, fire resistance and lightning strikes.

## Figures and Tables

**Table 1:** Kinetic parameters by Kissinger, Friedman and Ozawa methods.

Method	α	$E_{A}/(\mathrm{kJ}/\mathrm{mol})$	A/min^‒1^
Kissinger		264.671	3.088 × 10^24^
		216.508	3.14 × 10^13^
Friedman	0.2	211.957	1.343 × 10^14^
	0.3	215.000	
	0.4	217.153	
	0.5	222.009	
	0.6	221.851	
	0.7	285.503	
Ozawa	0.2	181.670	1.775 × 10^14^
	0.3	194.279	9.12 × 10^14^
	0.4	198.611	5.1 × 10^14^
	0.5	202.817	2.89 × 10^14^
	0.6	208.223	1.56 × 10^14^
	0.7	225.263	7.54 × 10^13^

**Table 2:** Kinetic parameters by Kissinger method.

Reactions		Step 1		Step 2	
Atmosphere		Ar	Air	Ar	Air
Activation energy	*E_A_*/(kJ·mol^−1^)	264.671	301.884	216.508	313.307
Pre-exponential factor	*A*/min^−1^	3.088 × 10^24^	1.96 × 10^28^	3.14 × 10^13^	1.75 × 10^21^
Order	n	0.94	0.93	0.96	0.945

**Table 3:** Thirteen kinds of commonly used pyrolysis kinetics models.

Mechanism	F(α)	Reaction Mechanism
F_1_	−ln(1−α)	One-dimensional random nuclear reaction
F_2_	11−α	Two-dimensional random nucleation reaction
F_3_	1(1−α)2	Three-dimensional random nuclear reaction
F_n_	$\left[{\left(1-\alpha \right)}^{1-n}-1\right]/\left(n-1\right)$	*n*th order
A_2_	${\left[-\ln\left(1-\alpha \right)\right]}^{1/2}$	Avrami random nucleation *n* = 2
A_3_	${\left[-\ln\left(1-\alpha \right)\right]}^{1/3}$	Avrami random nucleation *n* = 3
A_4_	${\left[-\ln\left(1-\alpha \right)\right]}^{1/4}$	Avrami random nucleation *n* = 4
R_2_	$1-{\left(1-\alpha \right)}^{1/2}$	Phase boundary reaction, cylindrical symmetry
R_3_	$1-{\left(1-\alpha \right)}^{1/3}$	Phase boundary reaction, spherical symmetry
D_1_	$\alpha^{2}$	One-dimensional diffusion
D_2_	(1−α)ln(1−α)+α	Two-dimensional diffusion, cylindrical symmetry, (Valensi equation)
D_3_	${\left[1-{\left(1-\alpha \right)}^{1/3}\right]}^{2}$	Three-dimensional diffusion, spherical symmetry Jander equation
D_4_	$\left(1-2\alpha /3\right)-{\left(1-\alpha \right)}^{2/3}$	Three-dimensional diffusion, spherical symmetry, Ginstling–Brounshtein equation

**Table 4:** Correlation coefficients corresponding to various mechanism function for the plot ln(F/T^2^)-1/T at different heating rates.

Activation Energy (kJ/mol)	β = 5 °C/min	β = 10 °C/min	β = 20 °C/min	β = 40 °C/min	R
F_1_	85.9009	90.2059	93.7321	97.1478	−0.98982
F_2_	46.0787	48.1592	50.0159	52.5525	−0.98913
F_3_	103.327	107.678	111.615	116.918	−0.99107
F_4_	199.137	207.792	215.374	224.461	−0.99922
A_2_	37.3654	39.4233	41.0745	42.6674	−0.98643
A_3_	21.1869	22.4958	23.52189	24.5073	−0.98121
A_4_	13.0976	14.0320	14.7456	15.4273	−0.97261
R_2_	72.4191	76.1849	79.2192	81.9901	−0.98259
R_3_	76.7291	80.6684	83.8604	86.8359	−0.98531
D_1_	132.311	139.064	144.484	149.150	−0.97677
D_2_	147.134	154.499	160.467	165.816	−0.98217
D_3_	164.628	172.696	179.304	185.485	−0.98728
D_4_	152.932	160.530	166.711	172.335	−0.98407

## Data Availability

The data presented in this study are available on request from the corresponding author.
